# Supplementary material for: Examining neighborhood-level hot and cold spots of food insecurity in relation to social vulnerability in Houston, Texas
Source: PLoS One. 2023 Mar 14;18(3):e0280620. doi: 10.1371/journal.pone.0280620 (PMC10013905; doi:10.1371/journal.pone.0280620)
Supplement: Attachment — Submitted filename: Response to Reviewers.docx [file pone.0280620.s001.docx]

Ryan Ramphul, PhD, MS

The University of Texas Health Science Center at Houston

Reuel Stallones Building

1200 Pressler St

Houston, TX 77030

(817) 874-2315

[ryan.ramphul@uth.tmc.edu](mailto:rramphul@gmail.com)

September 20, 2022

Dear Reviewers:

Thank you for your comments on my manuscript entitled “Examining Neighborhood-Level Hot and Cold Spots of Food Insecurity in Relation to Social Vulnerability in Houston, Texas.” I have edited the manuscript to address each point in the manner outlined below.

Sincerely,

Ryan Ramphul, PhD, MS

**Reviewer #1:** To know about neighborhood food insecurity authors geocoded food insecurity screening data provided by health provider, along with geospatial modeling, and Social Vulnerability Index. They found that hot spot tracts were having higher overall social vulnerability index scores, greater subdomain scores, and increased poverty, unemployment, limited English proficiency. Overall this is an interesting study. I think it is well written and properly explained but few minor suggestions are there to improve the paper:
When you say plethora of research …. and see last line in the para references provided same and only 1 in the whole paragraph. Better cite more literature here. See below
“A plethora of research suggests that food insecurity is not only highly prevalent but associated with several poor health outcomes. Regarding children, studies suggest that food insecurity is associated with an increased risk of birth defects, anemia, lower nutrient intake, cognitive problems, aggression, and anxiety (1). Research also indicates that food insecurity is associated with higher risks of children being hospitalized, having asthma, behavioral problems, depression, suicide ideation, poor oral health, and poor overall health (1). Regarding non-senior adults, studies show that food insecurity is associated with decreased nutrient intake, increased rates of mental health problems, diabetes, hypertension and hyperlipidemia, poor sleep outcomes, and poor overall health (1). Finally, studies indicate that food insecure seniors are more likely to be in poor health, depressed, and have limited daily activities compared to their food-secure peers (1).

- Thank you for this comment. I rewrote this paragraph to clarify that this information came from a systematic review of literature on the impact of food insecurity on health outcomes.

In introduction I think extra details on food security outcomes are given than needed. Instead of three paragraphs on outcomes better be concise.

- Thank you for this comment. I agree, I removed one of the paragraphs on the effects of food insecurity on health outcomes.

Last two Paragraphs of discussion section is about study limitation and strength, so better give a heading “The limitation and Strength.”

- Thank you for this comment. I added a “Strengths and Limitations” heading.

Literature and references are not sufficient give some more studies and literature.

- Thank you for this comment. In rewriting the manuscript based on the various comments provided, I ended up adding some references but removing others.

Reference 20 is in the conclusion. Better put these lines in discussion/analysis section.

- Thank you for this comment, I rearranged the discussion and conclusion sections to make them flow better. Also, I moved reference 20 from the conclusion section.

**Reviewer #2:** Thank you for allowing me to review this interesting manuscript called "Examining Neighborhood-Level Hot and Cold Spots of Food Insecurity In Relation to Social Vulnerability in Houston, Texas." It aimed to assess residential spatial patterns of Medicare and Medicaid beneficiaries who screened positive for food insecurity at health care locations in Harris County, Texas. Even though the study could contribute to the field, some aspects do not allow me to accept it in its current form.

The definition of food insecurity in the introduction lacks other essential elements such as food availability. Please, add the reference for the definition used.

- Thank you for this comment. Instead of paraphrasing the definition of food insecurity I updated the text with the USDA’s full definition of food insecurity, complete with information on the levels of food insecurity that they define.

2. I am not sure what the need for this study is. This should be stated clearly in the introduction.

- This is a good point. I revamped the introduction section of this paper and added a specific paragraph on aim of this study and why it is needed. See paragraph 3.

3. Please, clarify the aim of the study in the abstract and the text.

- This is a good point. I revamped the introduction section of this paper and added a specific paragraph on aim of this study and why it is needed. See paragraph 3.

4. The food insecurity information was collected during the pandemic. There is extensive evidence that food insecurity increased during the pandemic due to socioeconomic and health problems. How could this affect the results of this research? Is this a limitation of the study?

- This is an excellent point. Our food insecurity screening data was collected just before the onset of the pandemic, which ended up exacerbating food insecurity significantly. I added this point to the limitations section of this paper.

5. There are different levels of food insecurity. Why were they not considered in the analysis?

- This is an excellent point. I added some information in the Introduction section about how the USDA’s definition of food insecurity involves different levels of food insecurity. The reason our analysis does not break food insecurity into different levels is because we utilize the Hunger Vital Signs screening tool to screen for food insecurity. The Hunger Vital Signs tool is an adaption of the longer USDA U.S. Household Food Security Survey, that has been validated for effectiveness. Health care providers often use the Hunger Vital Signs tool because it is much easier to administer than the U.S. Household Food Security Survey, but unfortunately the Hunger Vital Signs tool only indicates if someone is food insecure or not. It doesn’t indicate if they are very food insecure.

6. I suggest adding the definition of hot and cold spots in the methods section and removing it from the results.

- Agreed, I added the definition of hot and cold spots to the methods section and removed it from the results.

7. The aim of the study should guide the conclusion. However, the authors conclude with ideas related to the healthcare system.

- This is a good point. I updated the Introduction section with a specific paragraph about the aim of the study and why it’s needed. I also rewrote the conclusion section to complement the aim paragraph, removing much of the text about the healthcare system.

8. In the discussion section, the authors state: "Researchers used to postulate that food-insecure people resided in "food deserts" until a growing body of evidence suggested otherwise." Here, it is essential to re-review the definition of food insecurity because it includes the lack of economic and physical access to food.

- Thank you for this point. I updated the Introduction section with the USDA’s full definition of food insecurity, complete with information on the levels of food insecurity that they define. I also updated paragraph 4 of the Introduction which discusses definitions of food deserts.

9. Please, review the names of all tables and figures.

- Thank you, I reviewed the names of all tables and figures and adjusted accordingly.

10. I suggest separating the results and discussion sections.

- Thank you, I separated the results and discussion sections**.**

**Reviewer #3**: This was an interesting article and thank you for the opportunity to review this article. I have suggested a few revisions that could further improve this paper.
List of comments/suggestions:

Abstract: Please include the sample size. If word count permits, consider providing the p values for the significant differences mentioned in the abstract.

- Thank you for this comment, I added sample size and p-values to the abstract.

Introduction: A citation should be provided for the statement “Food insecurity is highly prevalent in the US”. If possible, provide the most recent statistics on food insecurity prevalence. Consider defining food desert area when it is first mentioned on page 3. In the introduction the importance of specifically using the address-level food insecurity screening data and the spatial clustering methods.

- Thank you for these comments. I updated the statement about food insecurity prevalence, and added some updated statistics on the estimated number of households reporting food insecurity. I also added a paraphrased definition of “food deserts” when I first introduce the concept in the paragraph indicated. In addition, I rephrased the introduction section to stress the importance of using address-level food insecurity screening data and spatial clustering methods.

Materials and methods: This section could be clearer if you could break it into subsections under materials and methods. Provide a citation for the Hunger Vital SignTM screening tool. Consider using a flow diagram to show the participants' inclusion/exclusion. There is a typo on page 7, line 3. A citation is needed for the queen’s contiguity method. The second paragraph on page 7, requires a citation. Define the abbreviation SVI on page 8, the first line.

- Thank you for these comments, I separated out the Materials and Methods sections into two separate sections. I also added the citation for the Hunger Vital Sign Screening tool, and updated the typo (changed “than” to “then”) on page 7. In addition, I added a citation for the sentence about the queen’s contiguity method and added a citation for the second paragraph on page 7. Finally, I added text to indicate that SVI refers to the Social Vulnerability index in the first sentence of this paragraph.

Results and discussion:
I believe you have mistakenly used “… in terms of HPV vaccine uptake” in the title of Table 2. Also, there is a typo in this title. Include the Mann-Whitney U test statistics with the p-values in the table.
I think it will be better if you could add a footnote to the Table 2 indicating what this p-value is. It is in the text but missing from the table.

- Thank you for this comment. I corrected the title of the table, added median values and the test statistics, and also added a footnote indicating significance levels.

Conclusion: I feel that except for the final sentence other statements in this section could not be considered as conclusions based on this study's findings. Please include only the conclusions that are based on your findings.

- This is a good point. I rewrote the conclusion to focus on conclusions based on this study’s findings.

**Reviewer #4:** This paper provided a unique way to measure neighborhood food insecurity. There are some minor suggestions that would improve this paper:

Results and Discussion

Stating that finding the prevalence of food insecurity to be 54% in this study compared to Greater Houston at 16.6% is due to comfort with answering food insecurity screening in this setting might be an overstatement. People that attend this clinic were on Medicaid or Medicare, suggesting a higher probability of lower/fixed income.

Additionally, the method used to measure food insecurity in this study was the Hunger Vital Sign and the full USDA questionnaire may have been the method used to get the Greater Houston prevalence of food insecurity. Authors should report if this may have contributed to a difference in measurement for comparison.

- This is an excellent point about Medicaid and Medicare patients being on fixed incomes, I removed this section from the paper. As far as food insecurity rates in our sample versus Greater Houston, the 16.6% estimated food insecurity rate in Greater Houston comes from Feeding America’s food insecurity calculator, which estimates area-level food insecurity based on poverty rates and unemployment rates. Either way, I removed that section from the paper to avoid confusion.

The title for Table 2 does not make sense and appears to be copied from a different study.

- Thank you for this comment. I corrected the title of the table, added median values, test statistics, and added a footnote indicating significance level.
