# Supplementary material for: Examining neighborhood-level hot and cold spots of food insecurity in relation to social vulnerability in Houston, Texas
Source: PLoS One. 2023 Mar 14;18(3):e0280620. doi: 10.1371/journal.pone.0280620 (PMC10013905; doi:10.1371/journal.pone.0280620)
Supplement: Attachment — Submitted filename: Response to Reviewers.docx [file pone.0280620.s002.docx]

Ryan Ramphul, PhD, MS

The University of Texas Health Science Center at Houston

Reuel Stallones Building

1200 Pressler St

Houston, TX 77030

(817) 874-2315

[ryan.ramphul@uth.tmc.edu](mailto:rramphul@gmail.com)

December 29, 2022

Dear Reviewers:

Thank you for your comments on my manuscript entitled “Examining Neighborhood-Level Hot and Cold Spots of Food Insecurity in Relation to Social Vulnerability in Houston, Texas.” I edited the manuscript to address each point in the manner outlined below.

Sincerely,

Ryan Ramphul, PhD, MS

**Reviewer #1: Thank you very much for the responses. They have provided sufficient revisions. this can be accepted in its present form**

**Reviewer #2: Thank you for allowing me to review this new version of the manuscript titled "Examining Neighborhood-Level Hot and Cold Spots of Food Insecurity in Relation to Social Vulnerability in Houston, Texas." All my comments have been addressed. However, some minor aspects still do not allow me to approve this new version, mainly in the introduction section. The second paragraph indicates "a plethora of research..." and only one reference is cited in the text. I reviewed the answer to another reviewer's comment about this point. Even when the cited reference is a literature review, its use needs to be more coherent with the phrase at the beginning of the paragraph. The authors should use the original references or indicate "A literature review..." instead of "A plethora of research."**

- Thank you for pointing this out. I removed the phrase “A plethora of research” and clarified that this information came from a systematic review of literature on the impact of food insecurity on health outcomes.

**Please, revise all references cited in the text. When there are more than two references, the right way of citing is (3-6), not (3,4,5,6).**

- Thank you for this comment. I revised all references cited in the text. When there are more than two references, I cited as (3-6), not (3,4,5,6).

**Another limitation is that the study does not consider the different levels of Food insecurity. I suggest adding it.**

- Thank you, I added this point to my limitations paragraph.

**Reviewer #3: The authors have addressed most of the comments provided in the previous review. However, I have two minor comments that could improve this paper before publishing.**

**1. When reporting p values if the p =0.000 please indicate it as p< 0.001. In general p values smaller than 0.001 should be reported as p<0.001**

- Thank you for catching this, I changed to report all p values smaller than 0.001 as p<0.001.

**2. I think you can rearrange the materials and method section to make if flow better and easier to follow. e.g. The first paragraphs in material section and method section should come under the subheading "Data", as these two paragraphs explain the study sample or the data used. You could have another subheading "Food insecurity" for the paragraphs that explain the tool used for measuring food insecurity. All paragraphs explaining the statistical analysis conducted should be under the subheading "Statistical Analysis"**

- Thank you for this comment, I rearranged the materials and methods section the way you suggested, and it does indeed flow better.
